# Epidemiology of *Helicobacter pylori* Resistance to Antibiotics (A Narrative Review)

**DOI:** 10.3390/antibiotics12071184

**Published:** 2023-07-13

**Authors:** Irena Mladenova

**Affiliations:** Department of Hygiene, Epidemiology, Microbiology, Parasitology and Infectious Diseases, Medical Faculty, Trakia University, 6000 Stara Zagora, Bulgaria; imladenova@yahoo.com; Tel.: +359-897-324472

**Keywords:** *Helicobacter pylori*, epidemiology, antibiotic resistance, epidemiological studies, prevalence, geographic distribution, risk factors, emergency, resistance

## Abstract

*Helicobacter pylori* (*H. pylori*) is the most common bacterial infection worldwide and one of the main etiological factors of chronic gastritis, peptic ulcer disease, and stomach neoplasms. The mass application of antibiotics without testing, especially during the last years of the pandemic of SARS-CoV-2, could lead to a dramatic increase in antibiotic resistance and reduced effectiveness of eradication regimens for *H. pylori* infection. The epidemiology of *H. pylori* resistance to antibiotics still has unclear mechanisms. Antibiotic policy should be intensified to optimize treatment, and regular monitoring of resistance of *H. pylori* in different geographical regions should be conducted. Individualized treatment according to susceptibility testing is strongly advisable, and the best treatment regimens should be selected. The mutations in the genes encoding the antibiotic target protein are significant risk factors for *H. pylori* resistance. Iatrogenic errors in diagnosis and prescribing treatment for the failure of *H. pylori* eradication are other important risk factors. The low level of awareness and compliance with the correct treatment influence the rate of *H. pylori* resistance. Epidemiological surveillance of antibiotic resistance and the adoption of new treatment strategies are needed. The discovery of an efficient vaccine against *H. pylori* could reduce the pressure of the world’s growing antibiotic resistance.

## 1. Introduction

*Helicobacter pylori* is the most common bacterial infection worldwide and one of the main etiological factors of chronic gastritis, peptic ulcer disease, and stomach neoplasms (gastric carcinoma and MALT (mucosa-associated lymphatic tissue) lymphoma). The mass application of antibiotics without testing, especially during the last three years of the pandemic of SARS-CoV-2 (severe acute respiratory syndrome coronavirus 2), could lead to a dramatic increase in antibiotic resistance and reduced effectiveness of eradication regimens for *H. pylori* infection. The epidemiology of *H. pylori* resistance to antibiotics still has unclear mechanisms. There are few studies in the world literature on the epidemiology of *H. pylori*’s resistance to antibiotics. Studying the risk factors for antibiotic resistance can largely explain its mechanisms of distribution.

The objective of this narrative review was to track the latest research in the area of epidemiology of *H. pylori* resistance to antibiotics focusing on the geographic distribution and mainly on the risk factors (in the background of the pandemic during the last three years). In the review are included 60 studies.

## 2. Methods

This is a brief narrative review of the epidemiology of *H. pylori* resistance to antibiotics focusing on the most recent risk factors that have been studied and published in the last three years. The inclusion criteria were: original articles, meta-analysis, systematic reviews of geographic distribution and risk factors for *H. pylori* resistance to antibiotics published mainly over the last three years. The preprints, protocols, opinions, and publications with results without being definitive were excluded. The following keywords were used: “*Helicobacter pylori*”, “epidemiology”, “antibiotic resistance”, “prevalence”, “geographic distribution, “risk factors”, “epidemiological studies”, and “emergency resistance”, in English publications from the databases PubMed and PubMed Central. All data included in the review have been reported in PubMed-indexed scientific literature available online.

## 3. Epidemiological Surveillance of *Helicobacter pylori* Resistance

### 3.1. Geographic Distribution of Helicobacter pylori Resistance to Antibiotics

The prevalence of *H. pylori* positivity and antibiotic resistance, from a geospatial perspective was studied by Schubert et al. in Adelaide, South Australia (1998–2017). They used choropleth maps. The research demonstrates a heterogeneous distribution of *H. pylori* and its resistance to antibiotics. There was an association with migrant status. The migration status and geographic location need to be taken into account to select appropriate therapy for the eradication of *H. pylori* infection [1]. Data from Southeast China suggest that there was an increase in all resistance models (without the single resistance) between 2015–2017 and 2018–2020 in comparison with 2012–2014. The resistance to clarithromycin + metronidazole was significantly associated with age. The increase in the rate of resistance to clarithromycin and the triple resistance against clarithromycin, metronidazole, and levofloxacin, in children was associated with an *H. pylori* treatment in the past. Individualized treatment according to susceptibility testing is strongly advisable, and the best treatment regimens should be selected for *H. pylori* eradication [2]. One of twelve recommendations of a practice guideline for *H. pylori* eradication in China is in favor of the bismuth-containing regimen. They are cost-effective and efficient, especially for societies with limited resources with a similar high rate of antibiotic resistance as in China [3].

The rate of antibiotic resistance of *H. pylori* in a study by Boyanova et al. in people living in Sofia (the capital of Bulgaria) and those living in the country has been, respectively, 4.0 and 6.0% for amoxicillin, 48.0 and 42.0% for metronidazole, 30 and 30% for clarithromycin, and 4.0 and 4.0% for tetracycline. Resistance to levofloxacin has been 38.0% in the capital compared to 20.0% (*p* = 0.047) in those living elsewhere. The results suggest that the use of antibiotics should be controlled, and the unwarranted use of levofloxacin should be limited. Antibiotic policy should be intensified to optimize treatment, and regular monitoring of resistance of *H. pylori* in different geographical regions should be conducted [4]. In research by Fauzia et al., the mutations in the genes associated with antibiotic resistance of some clinical isolates from patients in Bangladesh have been studied. The results of that study suggest that the mutations in the genes encoding the antibiotic target protein are the main risk factors and mechanisms of *H. pylori* resistance [5]. The conclusion of one meta-analysis about the genotypic testing of clarithromycin resistance from fecal samples suggests that it is a correct, rapid, and non-invasive method, providing the accurate diagnosis of *H. pylori* clarithromycin resistance and guiding the reasonable choice of antibiotics [6].

Monoresistance of *H. pylori* to clarithromycin, metronidazole, or levofloxacin is very common. The prevalence of resistance to amoxicillin and tetracycline remains low. *H. pylori* infection, reinfection, or relapse pose serious challenges, and the choice of a successful *H. pylori* eradication regimen is a difficult decision [7]. In Colombia, South America, has one of the highest prevalence rates of *Helicobacter pylori* infection and stomach cancer in the world. The treatment efficacy is threatened by the emergence of antibiotic-resistant strains of *H. pylori*. Very interesting data showed no resistant strains against amoxicillin, clarithromycin, and rifampin (among 28 *H. pylori* strains from a high gastric carcinoma risk population and 31 strains from a low-risk population) [8].

In a retrospective cohort study from Zurich, Switzerland, the eradication regimen of *H. pylori* infection in 1721 patients was analyzed. The authors concluded that despite increasing the *H. pylori* resistance against clarithromycin in the world, there was no difference in the eradication success between the two main regimens: clarithromycin-containing versus non-clarithromycin schemes (71% and 71%, respectively) [9]. In total, 2735 *H. pylori* isolates from 873 patients were investigated (2003–2022) in Korea. An increased resistance rate was discovered against clarithromycin (16.1–31.0%), metronidazole (30.6–38.1%), and levofloxacin (7.3–35.7%). The progress of the multidrug resistance to clarithromycin and metronidazole (9.2–37.9%), clarithromycin, and fluoroquinolone has been very large and significant (2.8–41.7%, *p* < 0.001) [10]. The rate of resistance observed in one study by Mégraud et al. led to the conclusion not to proceed to an empirical triple therapy containing clarithromycin according to eradication therapy guidelines. Epidemiological surveillance of antibiotic resistance and the adoption of new treatment strategies are needed in the USA (United States of America) and Europe, especially in the context of the three-year pandemic SARS-CoV-2 [11].

The aim of another study was to research *H. pylori* resistance to metronidazole in Morocco and to investigate the influence of the virulence factors CagA (cytotoxin-associated gene A) and VacA (vacuolating cytotoxin A) on that resistance. There was no link with social factors. According to the virulence factors, the moderately virulent strains have been more resistant to metronidazole in comparison to the less virulent strains and highly virulent strains [12]. Essaidi et al. evaluated the link between epidemiological factors on resistance to some antibiotics in Morocco and found no connection. Their conclusion was in favor of the idea that each population should do its epidemiological studies and choose adequate eradication regimens according to the local antibiotic policy [13].

A meta-analysis of 248 articles suggests the conclusion that the overall rate of resistance to clarithromycin was 27.53% worldwide. The difference in the level of resistance could be the result of the antibiotic prescription rates in different geographical regions, the use of different imprecise criteria in conducted studies, or due to emerging multidrug-resistant strains [14]. Recently the resistance of *H. pylori* against amoxicillin has appeared to increase in Vietnam. It will affect the effectiveness of the eradication treatment. Tran et al. noted the emergence of new mutations significantly related to this antibiotic resistance [15]. A team of authors from Argentina has studied virulence factors of *H. pylori* and genotypes in patients with different histological findings [16].

Strains with less virulent genotypes have been found in Indonesia. Perhaps this is the reason for the low prevalence of *H. pylori* infection in respect to gastric cancer in comparison with the neighboring countries [17]. A 6-year surveillance has been performed in Israel. Clarithromycin and metronidazole resistance rates have been the highest- 46.3% and 16.3%, resp. It is noteworthy that multi-resistance has been statistically higher in Arab in comparison to Jewish patients [18].

The study (a meta-analysis) of multidrug resistance of *H. pylori* in children is the work of another team of authors who found a significant difference between Asian and Western countries [19]. A study by Antunes et al. reports a significant downward trend in the prevalence of *H. pylori* in childhood in Portugal, despite the high prevalence rate in other southern European countries [20]. Another meta-analysis of resistance patterns of *H. pylori* strains has been performed in the United States. The rate of resistance to metronidazole, levofloxacin, and clarithromycin was above 30%; therefore, choosing an empiric antibiotic regimen without cognition of the antibiotic resistance models is not acceptable [21].

In some Asian countries (Japan, China, Taiwan, and Korea), the prevalence of *H. pylori* infection and gastric carcinoma has recently increased based on the increased antibiotic resistance of the strains and the failure of main eradication regimens. Knowledge of molecular mechanisms of antibiotic resistance could help to develop new strategies [22]. Increasing resistance to metronidazole negatively affects the effectivity of *H. pylori* eradication, and an increase of the dose is recommended to overcome the high level of resistance [23,24,25]. The inclusion of nitazoxanide in the main clarithromycin-based treatment regimen leads to the success of eradication therapy [26].

The discovery of an efficient vaccine against *H. pylori* could reduce the pressure of the world’s growing antibiotic resistance. A recombinant type of *H. pylori* vaccine could be used for prophylactic and therapeutic vaccination [27].

### 3.2. Risk Factors for the Resistance of Helicobacter pylori to Antibiotics

A multivariate analysis of the risk factors for the resistance to five highly used antibiotics in Yangzhou, China, has been performed. The past clinical history and related outcomes have been significantly associated with *H. pylori*’s resistance against clarithromycin. Drinking, gastrointestinal complaints, and family anamnesis of gastric carcinoma have been associated with levofloxacin resistance [28]. In a cross-sectional study with 1042 online respondents in the USA, knowledge of *H. pylori* infection has been low, but difficulties in treatment have been high. The **low level of awareness and compliance** with the correct treatment are the main risk factors that influence the rate of *H. pylori* antibiotic resistance and the eventual development of gastric carcinoma or other forms of complications of *H. pylori* infection [29].

The study by another author group revealed the prevalence of mutations of *H. pylori* strains associated with resistance to clarithromycin, as well as of VacA and CagA virulence factors in infected Mexican patients. The **prevalence of mutations** associated with resistance to clarithromycin was more than 15%. Between the mutation A2142G and the vacA s1m1/cagA+ genotype, a significant association (*p* < 0.05) was found [30]. **Medical practice errors in diagnosis and prescribing eradication treatment** are another important risk factor for increased antibiotic resistance. In a retrospective study of 1730 patients in Spain, it was observed that despite the failure of first-line treatment, clarithromycin was repeated in 2.6% of patients on second-line treatment. Testing by gastroenterologists for *H. pylori* was missing in 2.5% of cases and is rare in the absence of an official indication [31]. Infectious agents (including *H. pylori*) are modifiable risk factors for the development of neoplasms. The regional antibiotic policy is a means of avoiding antibiotic resistance in cancer patients (with carcinomas, with a risk factor of bacterial infection, responding to antibiotic treatment). We should not forget the dual role played by bacteria—also as part of the beneficial microbiome [32].

Two hundred and fifty samples of meat products were examined in the city of Mansoura, Egypt. *H. pylori* was confirmed by PCR in 40.8% of raw meat products. Antimicrobial susceptibility testing revealed that all 110 molecularly confirmed *H. pylori* isolates were resistant to four or more antibiotics. The **contamination of meat products with multidrug-resistant *H. pylori*** may play an important role as a factor of transmission of *H. pylori* infection [33]. It is important to note that most of the expert consensus regarding *H. pylori* infection ignores **the elderly population** as a risk patient group. When choosing eradication regimens, a risk-benefit assessment should be made in the elderly, especially to reduce the risk of developing gastric carcinoma [34].

A multivariate analysis performed in Korea has shown that female gender, age over 50 years, **data for previous use of macrolides** as etiological treatment, and evidence of a recent respiratory infection have been significant risk factors for *H. pylori* resistance to clarithromycin [35]. The data from a cross-sectional observational study in Yunnan, China, showed that most people were resistant to antibiotics (276 patients were resistant to at minimum one antibiotic). Metronidazole and levofloxacin must no longer be the basic drugs for *H. pylori,* but amoxicillin should be prescribed as first-line treatment for *H. pylori* eradication. The unusual conclusion was that female gender, smoking, and drinking alcohol could be protective factors against metronidazole and rifampicin resistance [36].

**Iatrogenic risk factors** for the failure of *H. pylori* eradication in 508 patients have been studied by Xie et al. In 17.5%, one antibiotic with a high degree of regional resistance has been used incorrectly in triple therapy, and in 11.2%—two antibiotics or others unsuitable for quadruple therapy. In all, 178 schemes containing antibiotics with high levels of resistance have been used repeatedly in 85 patients [37].

Megraud et al., from the European *Helicobacter pylori* Antimicrobial Susceptibility Testing Working Group, have investigated the association between antibiotic consumption (2008–2017) in Europe and the resistance rates in the countries of the community. The conclusion is in favor of the statement that eradication therapy of *H. pylori* infection should not be initiated without **antibiotic susceptibility testing** [38].

Poor **compliance** is the subject of other studies. The use of eMedicine, which is online clinical medical knowledge or using smartphone applications, could improve compliance through higher-level development of the links between medical doctors and patients. The inclusion of probiotics to avoid the side effects of the eradication treatment, choosing longer courses of treatment (14 days leads to a better outcome than shorter—7–10 days), and matching the regimen with the patient’s previous antibiotic history are factors that can positively influence the success of the treatment of *H. pylori* infection [39,40].

The prevalence of *H. pylori* antibiotic resistance in HIV-positive patients is higher, according to a study by Nkuize et al. Multivariable regression analysis has shown that HIV (human immunodeficiency viruses) infection has been a risk factor for multiple antibiotic resistance of *H. pylori*. The authors concluded that monitoring of *H. pylori*’s antimicrobial susceptibility among HIV-positive subjects is necessary, and empiric treatments should be avoided [41]. Another team of researchers studied 258 HIV (+) individuals and 204 HIV (-) controls. The significant difference between the rates of eradication failure in patients and controls (24.1% versus 8.8%) is striking. The selected eradication regimen regarding *H. pylori* infection, **HIV (+) status, antiretroviral treatment, and alcohol consumption** are identified as risk factors for treatment failure. Coinfection of individuals with HIV and *H. pylori* is often associated with failure to eradicate *H. pylori* infection [42].

The aim of a prospective cohort study in southern China has been to investigate 22 potential geographic and **socioeconomic risk factors** for *H. pylori* antibiotic resistance. More than two eradication courses of treatment, more than three living in the same residence, and relatives with gastric cancer are risk factors for clarithromycin and metronidazole resistance of *H. pylori*. For the dual resistance of levofloxacin and metronidazole, **the age over 40 years, the low gross domestic product per capita, the greater number of doctors per 10,000 population,** as well as the higher human development index can be defined as risky [43]. Another study completed in Vietnam evaluated the risk factors for *H. pylori* antibiotic resistance among patients with chronic gastritis. The results have shown high levels of *H. pylori* resistance to clarithromycin and levofloxacin. After the multivariate analysis, **age over 30 years and previous eradication treatment** of *H. pylori* infection have been identified as risk factors for the resistance against clarithromycin, and age over 35 years has been a risk factor for levofloxacin resistance. The main conclusion that emerges from the study is that clarithromycin-based empiric eradication therapy should not be applied, and the inclusion of levofloxacin must necessarily be preceded **by susceptibility testing of *H. pylori* strains** [44].

Erkut et al. aimed to investigate the sociodemographic and clinical risk factors for the prevalence of *H. pylori* infection and antibiotic resistance in Turkey. Through logistic regression analysis, the authors found that the main risk factor for *H. pylori* resistance to **clarithromycin** has been **a history of its previous administration** (OR: 6.25, 95% CI: 1.59–24.52, *p* = 0.009). Choosing an adequate and correct eradication treatment is related to understanding and considering the risk factors for antibiotic resistance of *H. pylori* and can reduce the rate of failed regimens [45]. It is known that **clarithromycin-resistant polymorphisms of *H. pylori*** can be inherited and are a risk factor for failure of eradication treatment. Deguchi et al. investigated the risk of failure of clarithromycin-based triple therapy when present in the parents. The cross-sectional study included 404 individuals with a history of prescribed triple therapy (2005–2018). Multivariate analysis has found a significant association between failure of standard clarithromycin-based triple therapy for *H. pylori* eradication in parents and failure of this treatment in their children (OR 1.93; 95% CI—1.10–3.39). This identifies the **failure of classical triple therapy in parents** as a risk factor [46].

To determine the primary resistance of *H. pylori* strains and risk factors, 1851 positive patients were examined in Germany. The resistance to clarithromycin has been 11.3%, to levofloxacin—13.4%, to tetracycline—2.5%. Certain risk factors for resistance to clarithromycin have been **female sex, past antibacterial treatments, and advanced age over 65 years**—for resistance to levofloxacin. In Germany, clarithromycin-based triple therapy could be recommended as first-line, but strain susceptibility testing of antibiotic resistance is recommended [47]. Refractory *H. pylori* infection has been the subject of the study by Hanafy et al. 200 patients were treated with the standard triple therapy for two weeks. One hundred and thirty-six patients had a negative fecal antigen test, 15 had persistent dyspeptic symptoms and a positive test, and 49 had continued symptoms regardless of a negative fecal antigen test. Hidden predictors of refractory *Helicobacter* infection appear to be **diabetes mellitus, HCV (hepatitis C virus), and nonalcoholic fatty liver disease**, which require culture and antibiotic susceptibility testing of *H. pylori* strains [48].

The risk factors for the frequent failure of quadruple bismuth-based eradication therapy for *H. pylori* are not known. Multivariate analysis was performed regarding **compliance and duration of treatment, age over 60 years,** adverse reactions, and resistance to metronidazole have been independent risk factors for eradication treatment failure (*p* = 0.007) [49]. The results of another study showed that the mean level of 25[OH]D has been significantly lower in patients with failed eradication in comparison with the successful therapy group of individuals (14.7 ± 4.5 vs. 27.41 ± 7.1; *p* < 0.001). 25-OH vitamin D deficiency should be considered as a risk factor associated with failed eradication of *H. pylori* infection. A randomized trial is needed to determine the effect of vitamin D inclusion on *H. pylori* eradication [50].

Alaska has a high prevalence of *H. pylori* infection, antimicrobial-resistant strains of *H. pylori*, and gastric cancer. Risk factors for the carriage of antimicrobial-resistant *H. pylori* in 800 isolates were studied. **Female gender** has been a risk factor for metronidazole and clarithromycin resistance (*p* < 0.001 for both), and **urban residence** has been a risk factor for levofloxacin resistance (*p* = 0.003), while **advanced age** has been a risk factor for metronidazole and levofloxacin resistance (*p* < 0.05) [51]. A retrospective analysis of *H. pylori* isolates from children in Israel has been performed to determine primary and secondary antibiotic resistance. Among untreated children, primary total resistance has been 38% (to clarithromycin 9.5%, to metronidazole—32.6%, and to both 4.2%). If the children were treated, the rate of resistance has been, respectively: 71% (*p* = 0.002), to clarithromycin—29% (*p* = 0.02), and to metronidazole—61% (*p* = 0.007). Multivariate analysis has shown that **previous eradication treatment** was an independent risk factor for *H. pylori* antibiotic resistance in children in Israel. The result that all strains of *H. pylori* are susceptible to amoxicillin, tetracycline, and levofloxacin is striking. It is of particular importance that the prescribed treatment in previously treated children is based on cultural examination [52].

In another scientific study, multivariate logistic regression analysis found no statistically significant predictors. A trend toward statistical significance has been seen for *H. pylori*’s negative status and minimal plasmacytic differentiation (*p* = 0.078 and *p* = 0.09). Low-grade MALT lymphomas are among the few neoplasms that respond to medical treatment. Plasmacytic differentiation of MALT lymphomas is associated with the outcome of eradication treatment of *H. pylori* infection [53]. A prospective study has been conducted on HIV-positive and HIV-negative patients in order to compare the antibiotic resistance in them as well as the responsible risk factors. HIV-positive individuals tend to be infected with *H. pylori* resistant to levofloxacin, metronidazole, or multidrug-resistant strains. Ethnicity and HIV status have been identified as independent risk factors for *H. pylori* resistance. The prevalence of primary *H. pylori*-resistant strains is greater in HIV-positive compared to HIV-negative patients. **AIDS** (acquired immunodeficiency syndrome) **and sex** have been predictive factors of the studied *H. pylori* resistance [54]. In another review, White et al. have looked at some mainly clinical risk factors associated with *H. pylori* antibiotic resistance, such as **previous antibiotic treatment, advanced age, gender, ethnicity, race, excessive alcohol use, and diagnosed dyspepsia.** Consideration of these factors is essential for prescribing the most appropriate therapeutic regimen for the eradication of *H. pylori* infection [55,56,57,58,59,60].

## 4. Discussion

The mass application of antibiotics without testing, especially during the last three years of the pandemic of SARS-CoV-2, could lead to a dramatic increase in antibiotic resistance and reduced effectiveness of eradication regimens for *H. pylori* infection. Epidemiological surveillance of antibiotic resistance and the adoption of new treatment strategies are needed. The mutations in the genes encoding the antibiotic target proteins are the main risk factors of *H. pylori* resistance. Knowledge of molecular mechanisms of antibiotic resistance could help to develop new strategies. Hidden predictors of refractory *H. pylori* infection appear to be diabetes mellitus, HIV, HCV, and nonalcoholic fatty liver disease, which require culture and antibiotic susceptibility testing of *H. pylori* strains. Iatrogenic errors in diagnosis and prescribing treatment for the failure of *H. pylori* eradication are other important risk factors for increased antibiotic resistance. In patients with a tendency to develop antibiotic resistance, clinicians could prescribe a bismuth-based treatment or some of the more recently studied drugs against *H. pylori* infection, such as vonoprazan or nitazoxanide. The low level of awareness and compliance with the correct treatment influence the rate of *H. pylori* antibiotic resistance and the eventual development of gastric carcinoma or other forms of complications of the infection.

## 5. Conclusions

Each population in different geographical regions should conduct its epidemiological studies and choose adequate eradication regimens according to the local antibiotic policy. Individualized treatment according to susceptibility testing is strongly advisable. Local antibiotic resistance is monitored, but the information is not always up-to-date and reliable. A greater collection of demographic and clinical data as potential risk factors for antibiotic resistance through regular patient surveys may help to more accurately guide appropriate empiric treatment. Choosing an adequate and correct eradication treatment against *H. pylori* is related to understanding and considering the risk factors for antibiotic resistance of *H. pylori* and can reduce the rate of failed regimens. More studies are needed on the epidemiology of *H. pylori*’s resistance to antibiotics. The discovery of an efficient vaccine against *H. pylori* could reduce the pressure from the world’s growing antibiotic resistance.

## Data Availability

Not applicable.
